# Assessment of Potentially Unnecessary Breast Magnetic Resonance Imaging Utilization and Its Economic Impact: A Retrospective Study

**DOI:** 10.5152/eurasianjmed.2026.261562

**Published:** 2026-08-13

**Authors:** Düzgün Can Şenbil, Müslüm Burak Kılıçparlar, Mecit Kantarcı, Sonay Aydın

**Affiliations:** 1 Department of Radiology, Erzincan Binali Yıldırım University, Erzincan, Türkiye; 2 Department of Radiology, Atatürk University, Erzurum, Türkiye

**Keywords:** Breast cancer, economic impact, mammography, MRI, US

## Abstract

**Background:**

In this study, breast magnetic resonance imaging (MRI) examinations were retrospectively reviewed. This study evaluated whether the indications for breast MRI were consistent with the recommendations of the Breast Imaging Reporting and Data System (BI-RADS)atlas. Additionally, the study assessed whether prior ultrasound and mammography findings were sufficient for diagnosis. The aim was to identify potentially unnecessary breast MRI examinations and their associated financial burden.

**Methods:**

Breast MRI reports were compared with previous mammography and ultrasound reports. Patients for whom breast MRI provided no additional diagnostic benefit were identified, and the indications for MRI examination were analyzed.

**Results:**

Comparison of BI-RADS categories assigned on prior ultrasound and mammography with those assigned after breast MRI revealed that 321 (64.3%) of the 499 patients could have been adequately managed without breast MRI. In these patients, MRI findings did not alter clinical management or treatment decisions.

**Conclusion:**

Breast MRI is a costly imaging modality with limited accessibility in many healthcare settings. Therefore, its economic impact should be carefully considered when determining appropriate indications for its use. These findings suggest that the absence of restrictive criteria for breast MRI requests may contribute to substantial financial losses due to unnecessary examinations.

**Main Points**

- For patients whose ultrasound and mammography reports indicate no need for further investigation, performing an magnetic resonance imaging (MRI) for various reasons leads to unnecessary costs, and these costs are much higher than expected.
- It is extremely important to adhere to BI-RADS criteria and radiologist recommendations when performing breast MRI examinations.
- If MRIs are ordered without proper indication compliance, it creates significant costs and leads to unmanageable congestion in radiology clinics.

## Introduction

Mammary glands are sweat glands in the human body that develop from the ectoderm and produce milk.^1^ Breast cancer is the second leading cause of death in the world, following cardiovascular disorders.^2^ Breast cancer affects around 12.5% of women, according to studies conducted in the United States and Europe.^2^ This rate is even higher in developing and impoverished countries.^3^ Breast cancer mortality rates have begun to fall in recent years, particularly in Europe and North America, thanks to early diagnosis and cutting-edge treatment options.^4^

Early breast cancer diagnosis has a substantial impact on therapy and prognosis.^2^ Manual examination is the primary way to examine the breast, but 3 major imaging and screening methods are chosen. Mammography is the principal imaging tool used to evaluate the breast. Mammography sensitivity reduces in middle-aged patients with high breast density, and it cannot distinguish between benign and malignant tumors in some circumstances.^5^ Breast ultrasonography (US) is the second most commonly used modality for breast evaluation. The primary advantage of ultrasonography is that it is inexpensive and does not emit ionizing radiation. However, the operator’s skill and experience influence image quality. When technologies like Doppler and Power Doppler are added, its sensitivity grows even further.^6^ Magnetic resonance imaging (MRI) is the third approach used to evaluate the breast. Magnetic resonance imaging has great sensitivity for discriminating between benign and malignant tumors in a patient. However, due to its lower specificity, false positive results may arise. When combined with contrast-enhanced dynamic series, its sensitivity increases even further. However, it takes a long time for MRI acquisition and image preparation for MRI evaluation, and the cost is significant. Breast MRI should be preferred in selected high-risk situations.^7^

All published guidelines suggest that screening should begin earlier in patients who are at high risk of developing breast cancer. However, mammography sensitivity declines with increasing breast density at an early age. Breast MRI sensitivity is higher in these patients and is unaffected by breast density.^8^^,^^9^

The expense of imaging is one of the most prominent factors that puts pressure on healthcare services and raises their costs in radiological breast examinations. According to a study conducted in the United States, the projected cost of unneeded imaging procedures is between 200 and 250 billion US dollars.^10^^,^^11^ Overuse of imaging methods alters the balance between outcomes and expenses, reducing societal benefits dramatically. Outpatient treatment expenses are steadily rising due to the growing quantity of high-cost imaging modalities. However, the excessive use of tests that have not been demonstrated to help patients by studies in the literature and whose indications for use have not been thoroughly identified is dragging health systems into financial problems and is beginning to have harmful impacts on patient health.^12^ In research where imaging methods and diverse protocols are employed, and requests without a scientific basis are not examined, productivity has grown, overuse of services has decreased, and waste of resources has considerably decreased.^12^

When selecting an imaging modality for breast disorders, consider its cost effectiveness. Cost-effectiveness studies in the literature frequently evaluate the cost effectiveness in breast cancer screening.^13^^,^^14^ It has been established that screening using an MRI in breast cancer patients without genetic alterations is quite costly.^15^ Based on the review of the literature, the use of MRI in screening programs has been evaluated in terms of cost effectiveness. However, no studies have been found that investigate the economic burden of breast MRI use in routine clinical practice. In this study, the economic impact and resource utilization associated with breast MRI examinations performed in the clinic were evaluated by looking at the causes for the scans, as well as previous US and mammography reports in patients who had breast MRI imaging (Table 1).

## Material and Methods

This study was conducted with the approval of the Erzincan Binali Yıldırım University Clinical Research Ethics Committee. The ethics committee number of the study is 303105. The ethics committee acceptance date is October 13, 2023. Informed consent requirement was waived due to the retrospective nature of the study.

The study was conducted retrospectively with 520 patients between January 1, 2020 and January 1, 2022 at the Department of Radiology, Mengücek Gazi Training and Research Hospital, Erzincan Binali Yıldırım University Faculty of Medicine. Prior ultrasound and mammography examinations were retrospectively reviewed by 2 radiologists. In cases in which interpretation was uncertain, an additional review was performed by a senior radiologist with extensive experience in breast imaging. Reviewers had access to available MRI findings and histopathological results during the assessment process.

**Figure 1.**
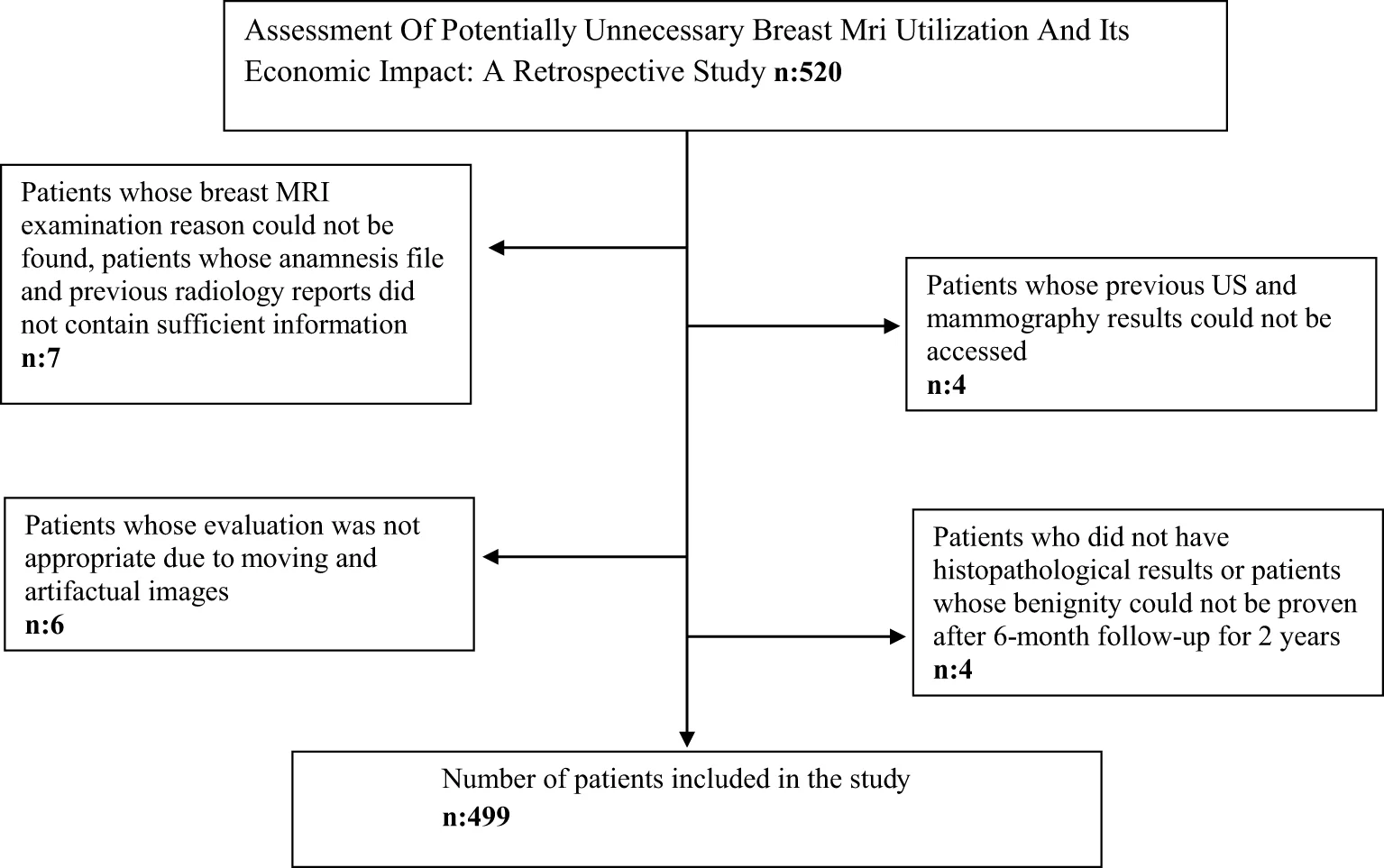
Flowchart of the study.

Patients whose indication for breast MRI could not be found, patients whose anamnesis file and previous radiology reports did not contain sufficient information, patients whose previous US and mammography results could not be accessed, patients whose evaluation was not appropriate due to motion or artifacts, patients who did not have histopathological results, or patients whose benign status could not be confirmed after 6-month follow-up for 2 years were not included. This study included patients who underwent breast MRI examination for any reason and whose picture quality was sufficient for review outside of the exclusion criteria, for a total of 499 patients (Figure 1). All consecutive patients who underwent breast MRI during the study period were screened for eligibility. Patients meeting the predefined exclusion criteria were excluded from the final analysis.

Breast MRIs were performed using 16-channel coils on a 32-channel MRI apparatus (Siemens Magnetom Aera, Erlangen, Germany) with a power of 1.5 Tesla. Patient images were analyzed using the Siemens Somatom Sensation-Syngo.via software program (Siemens Healthineers, Erlangen, Germany) from the Picture Archiving Communication System archive. The patients’ mammography, US, and MRI images were recorded using Akgün HBYS (hospital information management system) (Akgün software, Ankara, Türkiye).

All patients in the study were assigned a BI-RADS category based on the results of their breast MRI reports. Previous examinations were reviewed from the patient’s records, and US and mammography data were also documented. The patient’s US, Mammography (MG) results, and anamnesis data were evaluated to identify the indication for breast MRI and if it complied with breast MRI criteria. The evaluations examined whether any disease was missed during the breast MRI examination or if any pathology that differed from the documented mammography or ultrasound results was detected despite referral for further testing. Histopathology results were considered the gold standard for patient follow-up. For patients who did not have histology data, the gold standard was no cancer detection following a 2-year follow-up at 6-month intervals.

For the purposes of this study, potentially unnecessary breast MRI examinations were defined as examinations performed despite the absence of a recommendation for additional imaging based on prior ultrasound and/or mammography findings and in which MRI findings did not result in a change in diagnosis, treatment planning, or follow-up strategy. Imaging appropriateness was assessed according to BI-RADS-based recommendations documented in prior imaging reports and current guideline-supported indications for breast MRI. Because of the retrospective design, it is acknowledged that some patient-specific clinical factors may not have been fully captured in the available records.

According to the evaluated results, patients who were reported as benign or possibly benign based on mammography and ultrasonography results and for whom no further investigation was recommended, but who underwent breast MRI and revealed no additional pathology, were identified, and their proportion among the total study population was calculated. Furthermore, the economic burden of breast MRI exams performed at the facility was assessed using the MRI reimbursement fee specified in the Social Security Institution’s health practices circular.

The reasons for MRI scans of the patients are grouped under 7 groups in total and are listed as follows:

1. Patients who are requested to have an MRI due to the size of the lesion being over 3 cm.
2. Patients who underwent breast MRI despite benign ultrasound findings and no recommendation for further imaging, when medical records documented persistent patient concern or a request for additional diagnostic evaluation.
3. Patients who underwent breast MRI despite benign ultrasound findings and no recommendation for further imaging, based on the referring physician’s decision to obtain additional diagnostic evaluation.
4. Requesting an MRI for screening purposes in high-risk patients.
5. Previous examinations performed by the radiologist are not sufficient to make a decision, and the radiologist recommends breast MRI.
6. Requesting an MRI for staging purposes in patients diagnosed with malignancy.
7. Patients who underwent breast MRI following the presence of descriptive but non-suspicious terminology in ultrasound reports that prompted additional evaluation by the referring physician.

Magnetic resonance imaging indication categories were assigned retrospectively through a comprehensive review of referral forms, outpatient clinic notes, radiology reports, and documented clinical indications available in the hospital information system. Cases classified as patient-driven MRI requests were those in which the medical records documented persistent patient concern or a request for additional imaging despite benign imaging findings and the absence of a recommendation for further evaluation. Physician-initiated MRI requests were defined as examinations ordered despite benign imaging findings and without a recommendation for additional imaging in the original radiology report. Cases categorized as MRI requests triggered by report wording included examinations ordered following the presence of descriptive but non-suspicious terms in ultrasound reports. Examples included expressions such as “thin echogenic septa,” “slight lobulation,” “mild angular contour,” and “calcifications suggestive of milk of calcium.”

Artificial intelligence was not used in the writing, statistics, or publication stages of this article.

### Statistical Analysis

For statistical analyses, SPSS 25.0 (IBM Corporation, Armonk, New York, United States) program was used. For descriptive statistics, numerical variables are given as mean ± SD, min-max, and for categorical variables, number and percentage values are given. The relationship between the features at the qualitative measurement level was examined with Fisher’s exact test or Pearson chi-square test.

## Results

Our study included 497 female patients aged between 16 and 87 years, with an average age of 46.46 ± 12.04 years, and 2 male patients aged between 58 and 64 years, with an average age of 61 ± 4.24 years. The sociodemographic characteristics of the patients included in the present study are shown in Table 2.

Examining the reasons why patients had MRI exams, it was found that: 1 patient had an MRI because of a lesion larger than 3 cm; 15 patients had an MRI because of patient-driven MRI requests despite benign imaging findings; 135 patients had a breast MRI because of physician-initiated MRI requests despite benign imaging findings; 98 patients had a breast MRI for screening; 151 patients had a breast MRI at the radiologist’s recommendation; 44 patients had a breast MRI for staging because of a malignancy; and 55 patients had descriptive terms mentioned in the report (thin echogenic septa, a few slight lobulations, a slightly angular lesion, and calcifications suggestive of milk of calcium) (Table 3). Table 4 lists the reasons for breast MRI examinations.

When the BI-RADS results from the patients’ previous US and mammography results were compared to the BI-RADS grades after breast MRI, it was determined that 321 (64.3%) of 499 patients could be diagnosed without breast MRI and that breast MRI had no effect on the patient’s management or treatment. Breast MRI was shown to be necessary for diagnosing and treating 178 (35.7%) patients. Table 5 explains if the patients can be diagnosed based on their past results. In 178 individuals in whom MRI altered diagnosis or management, 2 had breast MRI despite being indicated for follow-up in US, and the subsequent biopsy revealed malignant tissue. In one of these patients, mammography was conducted prior to the US scan, and the outcome was BI-RADS 4. The lesion that could not be identified in the US was discovered in the second US scan following the MRI and was classified as BI-RADS 4. In the other patient, the mammography report was BI-RADS 0, thus US was advised. The patient’s US results were classified as fibrocystic change with calcification. Because the patient had been discharged from the nipple and the phrase calcification appeared in the report, the breast MRI result requested for the patient was recorded as BI-RADS 4. Histopathological examination confirmed malignancy. In the remaining 176 individuals, the previous breast US and MG indicated the necessity for advanced imaging. This circumstance was not determined to be statistically significant. In addition to these 2 patients, 6 more patients’ MRIs revealed lesions that were not mentioned in US or MG. These lesions, however, did not affect the patient’s BI-RADS category, treatment, or follow-up.

When a subgroup analysis was performed based on initial BI-RADS categories from conventional imaging, it was found that 68 patients with initial BI-RADS 2 findings underwent breast MRI. Regarding clinical management or follow-up protocol changes (indicating the clinical necessity of the examination), breast MRI influenced the management of 18 patients with initial BI-RADS 2, 44 patients with BI-RADS 3, 24 patients with BI-RADS 4, and 8 patients with BI-RADS 5. Additionally, among the patients with initial BI-RADS 3 findings, 18 patients were upgraded to a higher category (BI-RADS 4 or 5) following the breast MRI examination.

Breast MRI costs approximately $39.7 per patient based on the 2024 Health Implementation Circular (HIC) rates. Breast MRI is a contrast-based imaging technique, and the average cost of 15 mL of gadobutrol was calculated to be $37.3. The overall cost for a patient was calculated to be $77. In the present analysis, it was discovered that 321 patients did not require a breast MRI, and the total cost for these 321 patients was $24 526. This study covers a 2-year timeframe; hence, the annual cost was calculated to be $12 263.

## Discussion

Medicine, like other fields of science, is progressing rapidly in terms of technological breakthroughs and scientific investigations all over the world. This condition leads to the development of numerous diagnostic and treatment procedures in the healthcare sector. As a result of all of this, healthcare costs continue to rise unpredictably. Cost-cutting studies have become necessary as expenses have increased. However, the issue to remember when lowering expenses is not to sacrifice the quality and effectiveness of the operations to be implemented. Owens et al^16^ conducted a study in which they examined 3 basic principles. The first concept is to weigh the advantages, disadvantages, and costs of a test. According to the second notion, the cost of a test can be calculated by combining the cost of the test and the costs of its subtests. The third notion is the cost-effectiveness ratio, which calculates the extra cost required to gain additional health benefits. As a result, it provides a crucial assessment of the value of the examined health-related test.^17^

In the contemporary radiology literature, these challenges are increasingly addressed under the concept of “imaging stewardship.” Imaging stewardship emphasizes the responsibility of clinicians and radiologists to ensure evidence-based imaging utilization, thereby protecting patients from unnecessary procedures and optimizing healthcare spending. Implementing widely accepted radiology appropriateness criteria, such as the American College of Radiology Appropriateness Criteria or the BI-RADS atlas guidelines, serves as a vital gatekeeping mechanism. When advanced modalities like breast MRI are ordered without adherence to these established criteria, it directly undermines sustainable healthcare resource management. Effective imaging stewardship not only reduces the clinical workload and artificial congestion in radiology departments but also ensures that high-cost resources are preserved for patients who will derive definitive diagnostic or therapeutic benefit.

Data from clinical studies evaluating medications, medical devices, and treatment processes in medicine are increasingly being utilized to assess their economic value. The growing number of clinical studies now provides vital data for assessing cost-effectiveness. The increase in cost-effectiveness studies enhances the economic information available to assess developed technologies, and as a result, cost-effectiveness has emerged as a significant aspect in addition to clinical value when determining reimbursement. Cost-effectiveness data enables reimbursement institutions to make reimbursement and funding decisions in healthcare.^18^

The rising economic burden of chronic diseases and cancer has forced reimbursement institutions to prioritize cost-effectiveness alongside clinical efficacy to ensure healthcare sustainability. In a country where healthcare is primarily funded by government institutions, advanced diagnostic tools are heavily impacted by exchange rate fluctuations, as contrast agents and MRI equipment are mostly imported. Consequently, performing non-cost-effective tests that lack solid scientific support or clear patient benefit creates substantial waste. This study provides critical data on resource conservation by highlighting these unnecessary expenditures in general practice.^19^

The purpose of this study is to discover the indications for breast MRI requests, to compare the results of breast MRI to the patients’ past results, and to evaluate whether the diagnosis may be made using US and mammography findings. When breast MRI indications were reviewed, the main ones were found to be screening for high-risk women and assessing response to neoadjuvant chemotherapy.^20^ Apart from this, it is also used less frequently for research purposes and problem-solving role/purpose in cancers of unknown primary origin.^20^ When the reasons for breast MRI requests in this study are considered, it will be seen that they are not compatible with the reasons determined in the literature.^18^ These demands could be classified as problem-solving effects. However, there is some misunderstanding here. The problem-solving role/purpose is most commonly utilized in the BI-RADS vocabulary to investigate asymmetries observed in a single-exposure mammography. This condition is used to exclude the presence of a lesion due to the high negative predictive value of breast MRI.^21^ This study found that 65% of BI-RADS 2 and BI-RADS 3 patients were asked for a breast MRI. A comparison cannot be established because the literature contains little data on the MRI examinations requested by BI-RADS 2 and BI-RADS 3 patients. Several factors may contribute to increased MRI utilization, including diagnostic uncertainty, medicolegal considerations, patient expectations, and local practice patterns. However, these factors were not directly evaluated in the present study. According to the MRI results, the MRI reports of 2 patients previously classified as BI-RADS 2 were reclassified as BI-RADS 5. However, this is due to the radiologist’s inability to detect the BI-RADS 5 lesion. This cannot be considered a problem-solving role/purpose of breast MRI. In their study, Brady et al^22^ discovered that the error rate of radiologists ranged from 3% to 5%. Berlin et al^23^ reported in their study that radiologists’ mistake rates can be up to 30% when analyzed retrospectively. In the present analysis, the error rate in the reports is around 1.6%, which is lower than the mistake rate in the literature. As a result, it is believed that the findings obtained in MRI should not be interpreted as a problem-solving impact.

Studies on cost analysis in breast imaging are often conducted for imaging methods other than breast MRI.^23^ It was understood that reducing mammography screening from 2 years to 1 year for women with high-density breasts was not cost-effective and was abandoned.^15^ Again, in cost-effectiveness studies conducted on performing ultrasound on patients with no additional findings detected in mammography screening, it was decided that it was not cost-effective due to the small benefit it provided and the increased costs.^23^ In a study conducted by Lee et al,^20^ the combined use of tomosynthesis and mammography was evaluated and statistically significant results were obtained in terms of cost-effectiveness. Cost-effectiveness studies on breast MRI often focus on screening high-risk women.^20^^,^^25^^,^^26^ As far as the authors are concerned, no study in the literature has compared the cost effectiveness of breast MRI with the reason for the request. It is believe that this is related to the following. In other nations, particularly in Europe and North America, payment achieves cost-effectiveness by ensuring indication compliance. In the country, because indication compliance is not sought when imaging is performed, the cost-effectiveness phase is omitted. This research is significant because it demonstrates the impact of this predicament on the nation’s economy.

Although the unit cost of breast MRI in the country ($77) appears lower than international benchmarks—which range from $326 to $1432—the cumulative economic burden remains substantial. According to OECD(Organisation for Economic Co-operation and Development) data, the country has the highest MRI examination rate per 100 000 inhabitants despite ranking below the OECD average for the number of MRI units per million people (10.4 vs. 13.2). This high volume of scans on fewer machines accelerates equipment wear, compromises image quality, and escalates maintenance costs. Since the government finances most healthcare costs, reducing nonindicated scans is essential to mitigate this growing economic burden. ^14^^,^^27^^-^^29^

In this study, the mammography and ultrasound outcomes of patients who had a breast MRI before their scan were observed. Despite the report’s statement that advanced imaging technologies were not required for diagnosis, it was discovered that around 64% of patients received breast MRI. When the literature is reviewed, there is no study that is comparable to ours. The reason for this situation can be explained as follows: in developed countries, the BI-RADS dictionary recommendations are followed when performing scans, whereas in underdeveloped and developing countries, access to MRI devices is difficult, and insurance does not cover these costs. Several factors specific to the local healthcare landscape heavily contribute to this situation. First, the national healthcare policy and reimbursement system in the country (governed by the Social Security Institution) fully cover breast MRI examinations under the SUT without enforcing strict indication gateways or pre-authorization requirements. This lack of structural constraint, combined with high institutional imaging accessibility where patients can access advanced imaging free of charge, significantly lowers the threshold for ordering MRIs. Additional factors such as diagnostic uncertainty, medicolegal concerns, and institutional practice patterns may also influence MRI utilization; however, these factors were not specifically assessed in this study.

This research revealed that the cost per patient is $77. The annual loss due to unnecessary imaging was estimated at $12 263. Mertler et al^29^ discovered in their study that the number of medical equipment and imaging devices in Türkiye has increased over time. Since the increase in the number of medical devices will make MRI imaging more accessible, and there is no system to regulate demand, the aforementioned annual cost will increase exponentially over time.

This study has several limitations. First, its retrospective design may have limited the assessment of all clinical factors influencing MRI referrals. Additionally, prior imaging examinations were reassessed with knowledge of MRI findings and histopathological outcomes, introducing the potential for retrospective review bias. Second, this was a single-center study, which may limit the generalizability of the findings. Third, hospital admissions during the study period were affected by the COVID-19 pandemic and may have been lower than in other years, potentially leading to an underestimation of the annual economic burden. There have been numerous studies on the cost-effectiveness of breast MRI in high-risk women and those with negative mammogram results. However, to the best of the authors’ knowledge, no study has been conducted that reviews breast MRI scans retrospectively and compares patient results with indications to determine whether cost-effectiveness is considered when using breast MRI. This research is significant because it studies breast MRI images retrospectively in terms of economic impact.

In conclusion, in a substantial proportion of patients, breast MRI did not result in changes to diagnosis, treatment planning, or follow-up beyond those provided by conventional imaging. Adherence to established breast MRI indications and radiologist recommendations may help reduce unnecessary resource utilization and healthcare expenditures while maintaining appropriate patient care.

## Figures and Tables

**Table 1.** Current Guideline-Supported Indications for Breast Magnetic Resonance Imaging

Clinical Indication	Recommendation
Screening of women at high risk for breast cancer (e.g., BRCA1/2 mutation carriers)	Recommended
Evaluation of extent of disease in newly diagnosed breast cancer	Recommended in selected patients
Monitoring response to neoadjuvant chemotherapy	Recommended
Occult breast cancer presenting with axillary metastasis	Recommended
Problem-solving when mammography and ultrasound findings are inconclusive	Recommended
Silicone implant rupture assessment	Recommended
Routine screening of average-risk women	Not routinely recommended
Evaluation of probably benign lesions adequately characterized on conventional imaging	Generally not recommended
Additional evaluation of benign BI-RADS 2 findings without clinical concern	Not recommended

BRCA, breast cancer gene.

**Table 2.** Sociodemographic Characteristics of Patients

	n (%)
Gender	
Male	2 (0.4)
Female	497 (99.6)
Mean age ± SD (min-max)	46.48 ± 12.05 (16-87)
<35	83 (16.6)
35-50	252 (50.4)
50-65	133 (26.6)
>65	31 (6.2)

**Table 3.** Characteristics of Breast Magnetic Resonance Imaging Examinations Performed Due to the Word in the Report

	n (%)
Reason for MRI	
Contains thin echogenic septa	12 (21.8)
A few slight lobulations The lesion is slightly angular Calcifications suggest calcium milk	15 (27.2) 11(20) 17(30.9)

MRI, magnetic resonance imaging.

**Table 4.** Reasons for Patients to Undergo Magnetic Resonance Imaging

	n (%)
Reason for MRI	
Lesion size is over 3 cm	1 (0.2)
Patient-driven MRI request despite benign imaging findings Physician-initiated MRI request despite benign imaging findings For screening purposes in high-risk patients Inadequate diagnosis of previous examinations For staging purposes MRI request triggered by non-suspicious report wording	15 (3.0) 135 (27.0) 98 (19.6) 151 (30.2) 44 (8.8) 55 (11.0)

MRI, magnetic resonance imaging.

**Table 5.** Adequacy of Patients’ Previous Ultrasound and Mammography Results for Diagnosis

	n (%)
Could have been diagnosed with previous results	321 (64.3)
Could not have been diagnosed with previous results	178 (35.7)

## Data Availability

The data that support the findings of this study are available on request from the corresponding author.
